# Adaptive Sliding Mode Control for PMSM Drives with High-Order Disturbance Compensation

**DOI:** 10.3390/s26082402

**Published:** 2026-04-14

**Authors:** Bingmin Ji, Xin Mu, Lingbo Kong, Mingzhe Yang

**Affiliations:** 1 School of Electrical and Electronic Engineering, Changchun University of Technology, Changchun 130012, China; 18769370934@163.com; 2 Changchun Institute of Optics, Fine Mechanics and Physics, University of Chinese Academy of Sciences, Changchun 130022, China; 3 Electromechanical Research Laboratory, Chang Guang Satellite Technology Co., Ltd., Changchun 130000, China

**Keywords:** permanent magnet synchronous motor, adaptive reaching law, high-order disturbance observer, sliding mode control

## Abstract

To enhance the dynamic response and robustness of permanent magnet synchronous motor (PMSM) speed regulation under load disturbances, this study proposes a composite control strategy that integrates a novel sliding mode control based on an adaptive reaching law (NSMC) with a high-order disturbance observer (HDOB). First, an adaptive reaching law is designed to accelerate the convergence process when the system state is far from the sliding surface, while an adaptive saturation function (ASF) is introduced to smooth switching actions and reduce chattering near the sliding surface. Subsequently, a high-order disturbance observer is developed to estimate the lumped disturbance and its variation in real time, with the estimated disturbance being fed forward to the output of the speed-loop controller to enhance disturbance rejection capability. The effectiveness of the proposed method is validated through simulations and real-time experiments on a Hall-sensor-based PMSM drive platform. Experimental results show that, at a reference speed of 600 r/min, the proposed NSMC reduces settling time by 43.1% compared with conventional sliding mode control, while virtually eliminating overshoot. Under sudden load application and removal, the proposed NSMC + HDOB reduces the maximum speed deviation by 38.3% and 57.2%, respectively, compared with SMC + HDOB. These results indicate that the proposed strategy achieves faster speed tracking, smaller speed fluctuations, and enhanced robustness against load disturbances, offering an effective solution for high-performance PMSM drive systems.

## 1. Introduction

The permanent magnet synchronous motor (PMSM), characterized by high power density, high efficiency, and high-precision controllability, is widely applied in high-performance fields such as electric vehicle propulsion systems, industrial servo drives, and aerospace applications [1,2,3]. However, a PMSM is inherently a multivariable, nonlinear, and strongly coupled system, making it highly sensitive to parameter variations and external load disturbances [4,5]. These characteristics can lead to significant performance degradation of conventional model-based control strategies under certain operating conditions, thereby affecting control accuracy, dynamic response, and disturbance rejection capability.

To overcome these challenges, various advanced control methods have been developed. Nevertheless, due to their simple structure and ease of implementation under ideal modeling conditions, conventional control strategies remain widely adopted in PMSM control [6]. However, such methods typically rely heavily on accurate mathematical models of the controlled system [7]. In practical operation, when motor parameters vary or the system is subjected to unknown external disturbances, fixed controller parameters are often unable to maintain the desired dynamic and steady-state performance, resulting in reduced control accuracy and robustness. Consequently, in scenarios with complex dynamic conditions and stringent performance requirements, the applicability of conventional control strategies becomes inherently limited.

To address these limitations and improve control performance in high-precision applications, various advanced control strategies have been developed, including model predictive control (MPC) [8,9,10], adaptive control [11,12,13], active disturbance rejection control (ADRC) [14,15,16], and sliding mode control (SMC) [17,18,19].

The practical application of sliding mode control (SMC) faces two major challenges. First, to achieve its theoretical robustness, the controller parameters usually require careful and precise tuning. Second, the inherent chattering phenomenon of the system is difficult to eliminate completely. To mitigate chattering, continuous functions are commonly used to replace the discontinuous sign function, such as hyperbolic tangent functions and saturation functions, which provide smoother control signals [20,21,22]. The design of the reaching law plays a critical role in SMC performance, as it directly determines the dynamic process by which the system states converge to the sliding surface. This process is a core factor affecting system robustness. It should be noted that system robustness is relatively weak during the reaching phase; therefore, shortening the reaching time and accelerating convergence to the sliding surface are key objectives. An appropriately designed reaching law not only suppresses chattering but also significantly enhances overall system robustness. In recent studies, novel reaching laws have been proposed to effectively improve dynamic performance, including chattering suppression and robustness enhancement [23,24,25]. In conventional SMC, large switching gains are often selected to suppress disturbances. However, in practical applications, excessively large switching gains introduce severe chattering, which in turn degrades system robustness. To resolve this contradiction, disturbance observers have been introduced to estimate system disturbances, and the estimated disturbance is then compensated within the control loop [26,27,28]. Through disturbance observation and feedforward compensation, the influence of external disturbances can be effectively reduced, while the sources of chattering are simultaneously mitigated.

**Figure 1:**
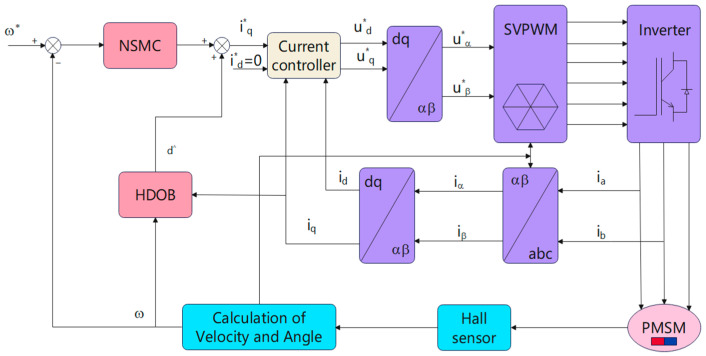
Schematic diagram of NSMC + HDOB control strategy.

The main contributions of this paper are summarized as follows:

(1) A novel adaptive reaching law is proposed for PMSM speed control. Different from conventional exponential reaching laws with fixed convergence characteristics, the proposed law automatically adjusts the approaching speed according to the distance between the system state and the sliding surface. As a result, rapid convergence is achieved when the state is far from the sliding surface, while the reaching speed is reduced near the equilibrium point to suppress chattering.
(2) An adaptive saturation function (ASF) is introduced into the reaching law to smooth the switching action near the sliding surface. This provides an additional mechanism for chattering attenuation without significantly weakening the robustness of sliding mode control.
(3) A high-order disturbance observer is designed to estimate the load disturbance and its variation in real time. By incorporating the estimated disturbance into feedforward compensation, the active disturbance rejection capability of the PMSM drive system is further improved.
(4) Comprehensive simulations and experiments, including speed tracking, sudden load disturbance, parameter variation, and q-axis current response analysis, are provided to verify the effectiveness and practical feasibility of the proposed control strategy. The structural block diagram is shown in Figure 1.

## 2. Design and Analysis of a Novel Fast Sliding Mode Reaching Law

### 2.1. Conventional Reaching Law

To facilitate the subsequent derivation, let s denote the sliding variable and $\dot{s}$ its time derivative. In the following performance analysis, the sliding surface is selected as:

(1) $$s=ce+\dot{e}$$

where e is the tracking error, with the unit of rad, and c > 0 is a constant representing the sliding-surface coefficient. Therefore, the unit of s is rad/s.

The reaching law plays a key role in sliding mode control (SMC) design, as it determines how the system state moves from outside the sliding surface toward it. During this reaching phase, the system error cannot be directly regulated, and the response is highly vulnerable to internal parameter uncertainties and external disturbances. Therefore, shortening this phase is crucial. Increasing the reaching law gain accelerates convergence to the sliding surface and enables faster stabilization. Consequently, designing an appropriate reaching law is essential for improving response speed, control quality, overall system performance, and robustness. The exponential reaching law, one of the most widely used forms, is expressed as follows:

(2) $$\dot{s}=-\varepsilon sgn(s)-qs$$

In this expression, sgn(s) denotes the sign function, with ε > 0 representing the switching gain and q > 0 the gain of the linear convergence term. The term −εsgn(s) guarantees that the system state reaches the vicinity of the sliding surface within a finite time, while −qs serves as the exponential convergence term, which accelerates the reaching speed and provides a damping effect when the sliding variable s is large.

According to Equation (2), the time required for the system to reach the sliding surface s = 0 from an initial sliding variable value s_0_ can be expressed as:

(3) $$t_{rh}=\frac{1}{q}ln\left(1+\frac{q\left|s_{0}\right|}{\varepsilon }\right)$$

When the system is far from the sliding surface (|s| is large), the reaching dynamics are primarily dominated by the term −εsgn(s), which drives the system to rapidly approach the sliding surface. This feature effectively shortens the system response time and provides a significant advantage in control scenarios that demand fast dynamic response.

When the system approaches the sliding surface (|s| is small), excessive reliance on the −qs term (a large q) combined with a relatively small discontinuous gain ε can result in insufficient reaching force near the sliding surface (|s| ≈ 0). This leads to slow convergence of the system states, prolonging the reaching time and reducing the overall response speed. More importantly, the inherent discontinuous switching behavior of the sign function sgn(s) near s = 0 remains the primary source of high-frequency chattering, which can degrade control accuracy and adversely affect actuator lifespan.

### 2.2. Adaptive Reaching Law

To address the aforementioned issues, an adaptive reaching law is designed, which can be expressed as follows:

(4) $$\left\{\begin{array}{c} \dot{s}=-(\frac{\varepsilon }{d\left(s\right)}+k{\left|s\right|}^{\alpha })ASF\left(s\right)-qs\\ d\left(s\right)=\lambda +\left(1+\lambda \right)\frac{1}{\cosh\left(\sqrt{\left|s\right|}\right)} \end{array}\right.$$

In the equation, ε > 0 is the switching gain of the reaching law, q > 0 is the gain of the linear convergence term, k > 0 is the gain of the nonlinear term, 0 < α < 1 is the power exponent, and 0 < λ < 1 is the shaping factor of the adaptive function. Both α and λ are dimensionless parameters. ASF(s) is the adaptive saturation function used to reduce chattering caused by the frequent switching of the sign function, and its expression is given as follows:

(5) $$\left\{\begin{array}{c} ASF\left(s\right)=\frac{s}{\left|s\right|+\delta \left(s\right)}\\ \delta (s)=\delta_{0}\cdot e^{-\eta \left|s\right|} \end{array}\right.$$

**Figure 2:**
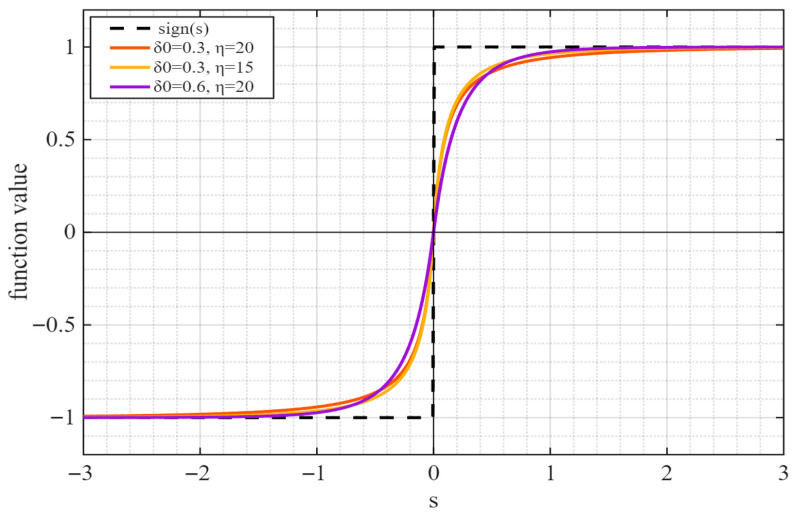
Comparison of ASFs with different slopes and the sign function sgn(s).

In the equation, $\delta_{0}$ is the basic smoothing factor, which is used to control the degree of smoothness in the small-error region; η > 0 is the error attenuation coefficient, which is used to regulate the smoothing factor as ∣s∣ increases. When s approaches zero, ASF(s) approximates a linear function $\frac{s}{\delta_{0}}$ with a slope of $\frac{1}{\delta_{0}}$. The function is continuous and smooth, which reduces system chattering. As |s| approaches infinity, ASF(s) asymptotically converges to the sign function sgn(s). This design preserves the inherent robustness of sliding mode control, ensuring that the system can effectively reject disturbances. The function is illustrated in Figure 2.

From the proposed adaptive reaching law, it can be observed that $\delta_{0}$ controls the smoothness of the function near s = 0; the smaller the value, the closer the function behaves to the sign function in the vicinity of zero. The parameter η regulates the transition speed of the function from the smooth region to the steep region; larger values result in a faster transition.

The core advantage of the proposed adaptive reaching law lies in its ability to adjust the convergence speed according to the system state. When the system state is far from the sliding surface (|s| is large), the term $k{\left|s\right|}^{\alpha }$ dominates. Combined with d(s) ≈ $\frac{1}{\lambda }$, the overall reaching speed $\frac{\varepsilon }{\lambda }+k{\left|s\right|}^{\alpha }$ is significantly increased, thereby accelerating the initial-phase dynamics of the reaching process and addressing the insufficient initial convergence speed observed in conventional reaching laws.

When the system state approaches the sliding surface (|s| → 0), the term $k{\left|s\right|}^{\alpha }$ decays synchronously with |s|, and d(s) approaches 1, causing the overall reaching speed to automatically decrease to approximately ε. This significantly reduces the kinetic energy and velocity of the system states as they cross the sliding surface. Simultaneously, the adaptive saturation function ASF(s), which exhibits a continuous linear characteristic near s ≈ 0, facilitates a smooth transition from the reaching phase to the sliding phase. This mechanism avoids the severe chattering caused by high-speed switching near the equilibrium point in conventional discontinuous control based on the sgn(s) function, thereby effectively suppressing chattering while preserving finite-time convergence.

### 2.3. Stability Analysis of the Novel Reaching Law

Considering the Lyapunov function shown in Equation (4):

(6) $$V(s)=\frac{1}{2}s^{2}$$

Taking the derivative of V(s) yields:

(7) $$\dot{V}(s)=s\dot{s}$$

(8) $$\dot{V}(s)=s\left[-(\frac{\varepsilon }{d(s)}+k{\left|s\right|}^{\alpha })ASF(s)-qs\right]$$

Based on the defined ASF(s) function, it follows that:

(9) $$s\cdot ASF(s)=\frac{s^{2}}{\left|s\right|+\delta (s)}=\frac{{\left|s\right|}^{2}}{\left|s\right|+\delta_{0}e^{-\eta \left|s\right|}}\geq 0$$

When s = 0, the equality holds. For s ≠ 0, it follows that s·ASF(s)>0.

Since 0 < λ < 1 and cosh($\sqrt{\left|s\right|}\geq 1$), it can be further derived that:

(10) $$d(s)=\lambda +\left(1-\lambda \right)\frac{1}{cosh\left(\sqrt{\left|s\right|}\right)}$$

$$\geq \lambda +\left(1-\lambda \right)\cdot 1=1>0$$

Therefore, $\frac{\varepsilon }{d(s)}>0$ holds for all s.

Substituting into the expression of $\dot{V}(s)$ yields:

(11) $$\dot{V}(s)=-(\frac{\varepsilon }{d(s)}+k{\left|s\right|}^{\alpha })s\cdot ASF(s)-qs^{2}$$

Since $\frac{\varepsilon }{d(s)}>0$, $k{\left|s\right|}^{\alpha }$ > 0, and s·ASF(s)≥0, it follows that:

(12) $$(\frac{\varepsilon }{d(s)}+k{\left|s\right|}^{\alpha })s\cdot ASF(s)\geq 0$$

Moreover, since $qs^{2}\geq 0$, it follows that:

(13) $$\dot{V}(s)\leq 0$$

The Lyapunov function satisfies $\dot{V}(s)=0$ if and only if s = 0, and $\dot{V}(s)<0$ strictly holds for all s ≠ 0.

According to the Lyapunov stability theorem, the system is therefore globally asymptotically stable.

### 2.4. Performance Analysis of the Reaching Law

To verify the performance of the proposed adaptive reaching law, a comparative analysis is conducted between the conventional exponential reaching law and the proposed adaptive reaching law under identical parameter conditions. The following controlled plant is considered:

(14) $$\ddot{\theta }(t)=-f(\theta ,t)+bu(t)$$

Among them, u(t) is the auxiliary control input of the second-order test system, used solely to verify the dynamic performance of the reaching law, and is not the actual control quantity in the PMSM system, $f(\theta ,t)=25\dot{\theta }$, b = 133;

The sliding surface is defined as follows:

(15) $$s=ce+\dot{e}$$

where c > 0.

Defining the desired position signal $\theta_{d}(t)$, the tracking error can be expressed as:

(16) $$\left\{\begin{array}{c} e=\theta_{d}(t)-\theta (t)\\ \dot{e}=\dot{\theta_{d}}(t)-\dot{\theta }(t) \end{array}\right.$$

Taking the derivative of s yields:

(17) $$\dot{s}=\dot{ce}+\ddot{e}=c(\dot{\theta_{d}}(t)-\dot{\theta }(t))+\left[\ddot{\theta_{d}(t)}+f(\theta ,t)-bu(t)\right]$$

By substituting the proposed adaptive reaching law into the above equation, the resulting control input can be expressed as follows:

(18) $$u\left(t\right)=\frac{1}{b}\left[(\frac{\varepsilon }{d(s)}+k{\left|s\right|}^{\alpha })ASF(s)+qs+c\left(\dot{\theta_{d}}(t)-\dot{\theta }(t)\right)+\left(\ddot{\theta_{d}(t)}+f(\theta ,t)\right)\right]$$

**Figure 3:**
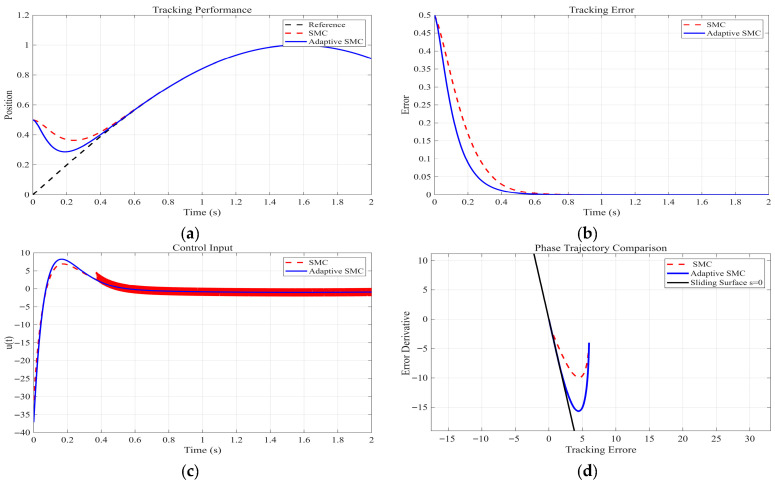
Performance comparison of the two reaching laws. (**a**) Tracking performance of the exponential reaching law and the adaptive reaching law. (**b**) Tracking errors of the exponential reaching law and the adaptive reaching law. (**c**) Controller outputs of the exponential reaching law and the adaptive reaching law. (**d**) Phase trajectories of the exponential reaching law and the adaptive reaching law.

The proposed adaptive reaching law is validated through MATLAB R2025a simulations with the following parameter settings: ε=0.1, k = 0.7, α=0.9, q = 20, λ=0.01, $\delta_{0}=0.1$, η=30, c = 1, and the desired position signal $\theta_{d}(t)$ = sin (t). The simulation results are presented in Figure 3.

Based on the comparison results shown in Figure 3a–d between the conventional exponential reaching law and the proposed adaptive reaching law, the following conclusions can be drawn:

Tracking performance (Figure 3a): The proposed adaptive reaching law achieves a faster initial response to the sinusoidal reference signal, demonstrating superior tracking accuracy and dynamic consistency.

Tracking error (Figure 3b): The conventional reaching law exhibits larger error amplitudes and slower convergence, whereas the adaptive reaching law significantly reduces the error magnitude and converges to near zero within a shorter time, indicating faster convergence and smaller steady-state error.

Controller output (Figure 3c): The conventional reaching law shows evident high-frequency chattering with large amplitude fluctuations. In contrast, the output under the adaptive reaching law is smooth, with chattering effectively suppressed, demonstrating the effectiveness of the ASF in mitigating chattering.

Phase trajectory (Figure 3d): The phase trajectory under the adaptive reaching law converges more quickly and smoothly to the sliding surface, indicating faster reaching speed and improved smoothness.

In summary, the results in Figure 3 clearly show that the proposed adaptive reaching law outperforms the conventional exponential reaching law in terms of tracking accuracy, convergence speed, chattering suppression, and smooth approach to the sliding surface. To resolve the trade-off between rapid convergence and chattering reduction, the proposed reaching law features an adaptive power term that remains large when the system state is far from the sliding surface to ensure fast convergence, and gradually decreases to zero as the state approaches the surface. In this way, the proposed method effectively overcomes the inherent conflict between convergence speed and chattering suppression found in conventional exponential reaching laws.

## 3. Design of the Sliding Mode Controller

### 3.1. PMSM System Model

The permanent magnet synchronous motor (PMSM) is a strongly coupled and complex nonlinear system, making the selection of an appropriate mathematical model essential. For the purpose of controller design, taking a surface-mounted PMSM as an example, the mathematical model in the d-q coordinate frame can be expressed as follows:

(19) $$\left\{\begin{array}{c} u_{d}=R_{s}i_{d}+L_{d}\frac{di_{d}}{dt}-p_{n}\omega_{m}L_{q}i_{q}\\ u_{q}=R_{s}i_{q}+L_{q}\frac{di_{q}}{dt}+p_{n}\omega_{m}(\varphi_{f}+L_{q}i_{q}) \end{array}\right.$$

In the above equations, $i_{d},u_{d}{,\;i}_{q}{,\;u}_{q}$ represent the stator currents and voltages along the d and q-axes, respectively. $R_{S}$ denotes the stator resistance, $p_{n}$ is the number of pole pairs, $\omega_{m}$ is the electrical angular velocity, $\varphi_{f}$ is the permanent magnet flux linkage, and $L_{d}$ and $L_{q}$ are the stator inductances along the d and q-axes, respectively.

### 3.2. Design of the Velocity-Loop Sliding Mode Controller

The velocity-loop controller is primarily designed to regulate the system’s speed output, ensuring that the system maintains good stability and accuracy even under external disturbances, parameter variations, and uncertainties. It is particularly applicable in scenarios requiring precise speed control and strong robustness.

To facilitate a better understanding of the design of the velocity-loop sliding mode controller, the PMSM system state variables are defined as follows:

(20) $$\left\{\begin{array}{c} e_{1}=\omega_{ref}-\omega_{m}\\ e_{2}=\dot{e_{1}} \end{array}\right.$$

Here, $x_{1}$ and $x_{2}$ represent the state variables of the sliding mode controller. $\omega_{ref}$ denotes the reference mechanical angular velocity, and $\omega_{m}$ is the actual mechanical angular velocity.

The choice of the sliding surface has a significant impact on the performance of sliding mode control. Linear sliding surfaces are widely used in the control of linear systems due to their simple design and analysis, strong robustness, and the ability to ensure favorable dynamic performance. Their advantages include ease of implementation and tuning, as well as good adaptability to various uncertainties and external disturbances. Therefore, a linear sliding surface is selected, which is expressed as follows:

(21) $$s=ce_{1}+e_{2}$$

In the equation, c > 0 is a design parameter.

Taking the derivative of both sides of the equation yields:

(22) $$\dot{s}=c{\dot{e}}_{1}+{\dot{e}}_{2}=ce_{2}-Du$$

where u is the output of the speed-loop controller, and D is the input gain of the motor mechanical system.

To ensure favorable dynamic performance of the three-phase PMSM drive system, the proposed adaptive reaching law is incorporated, yielding the following expression for the controller:

(23) $$u=\frac{1}{D}\left[ce_{2}+(\frac{\varepsilon }{d(s)}+k{\left|s\right|}^{\alpha })ASF(s)+qs\right]$$

Consequently, the reference current along the q-axis can be expressed as:

(24) $${iq}^{*}=\frac{1}{D}\int_{0}^{t}\left[ce_{2}+(\frac{\varepsilon }{d(s)}+k{\left|s\right|}^{\alpha })ASF(s)+qs\right]dt$$

The above expression represents the output of the velocity-loop controller designed based on the adaptive reaching law.

### 3.3. Stability Analysis of NSMC

To analyze the stability of the proposed adaptive sliding mode controller, the following Lyapunov function is selected:

(25) $$V(s)=\frac{1}{2}s^{2}$$

Taking the derivative of V(s) yields:

(26) $$\dot{V}(s)=s\dot{s}$$

By substituting Equations into the above expression, we obtain:

(27) $$\dot{V}(s)=s\dot{s}=s\left[-(\frac{\varepsilon }{d(s)}+k{\left|s\right|}^{\alpha })ASF(s)-qs\right]$$

An auxiliary function is defined as follows:

(28) $$\beta \left(s\right)=\frac{s^{2}}{\left|s\right|+\delta (s)}$$

It then follows that:

(29) $$\dot{V}(s)=-(\frac{\varepsilon }{d(s)}+k{\left|s\right|}^{\alpha })\beta \left(s\right)-qs^{2}$$

It can be derived that:

(30) $$\dot{V}(s)\leq 0$$

The Lyapunov function satisfies $\dot{V}(s)=0$ if and only if s = 0 and $\dot{V}(s)<0$ for all s ≠ 0. According to the Lyapunov stability theorem, the system is globally asymptotically stable, implying that the system states converge to the sliding surface from any arbitrary initial condition.

### 3.4. Design of the High-Order Disturbance Observer

To enhance the dynamic response and disturbance rejection performance of the motor in practical operation, particularly under external load disturbances, it is necessary to compensate for the performance degradation caused by such perturbations. Reference [29] presents various disturbance observation methods, among which high-order disturbance observers (HDOB) provide more accurate and faster estimation and compensation of time-varying disturbances and uncertainties, thereby comprehensively improving system performance in terms of precision, speed, robustness, and stability. In this study, the HDOB is applied to the permanent magnet synchronous motor (PMSM) to estimate external disturbances. The observed load torque disturbance is converted into a current compensation term, which is then fed forward into the system to enhance disturbance rejection.

For the PMSM speed control system, the mechanical motion equation can be expressed as:

(31) $${\dot{\omega }}_{e}=\frac{k_{t}P_{n}}{J}i_{q}-\frac{P_{n}}{J}T_{d}$$

In the above equation, ω_e_ denotes the electrical angular velocity of the motor, k_t_ is the torque coefficient, J is the moment of inertia, $p_{n}$ is the number of pole pairs, i_q_ is the q-axis current, and T_d_ represents the equivalent load torque disturbance. Here, the torque coefficient is given by $K_{t}=\frac{3p_{n}\varphi_{f}}{2}$.

To accurately estimate the disturbance, the load torque is modeled as a dynamic process. It is assumed that the disturbance torque varies according to a second-order dynamic model:

(32) $$\left\{\begin{array}{c} x_{1}=\omega_{e}\\ x_{2}=T_{d}\\ x_{3}=\frac{dT_{d}}{dt} \end{array}\right.$$

Based on the physical model, it can be expressed as:

(33) $$\frac{dx_{1}}{dt}=\frac{k_{t}P_{n}}{J}i_{q}-\frac{P_{n}}{J}x_{2}$$

It is assumed that the disturbance torque variation satisfies the following relationship:

(34) $$\frac{dx_{2}}{dt}=x_{3}$$

For the second-order disturbance observer, it is assumed that the rate of change in the disturbance torque is constant or varies slowly:

(35) $$\frac{dx_{3}}{dt}=0$$

This assumption indicates that when the disturbance variation rate is constant or slowly varying, it can adequately capture the characteristics of most mechanical load disturbances, while avoiding the amplification of noise that may result from high-order differentiation.

The complete state-space equation is given as follows:

(36) $$\left\{\begin{array}{c} \frac{dx_{1}}{dt}=\frac{k_{t}p_{n}}{J}i_{q}-\frac{p_{n}}{J}x_{2}\\ \frac{dx_{2}}{dt}=x_{3}\\ \frac{dx_{3}}{dt}=0 \end{array}\right.$$

### 3.5. Disturbance Observer Structure Design

Based on the above assumptions regarding external disturbances, an observer dynamic model consistent with the system dynamics is constructed. Output error feedback is introduced to correct the estimation process and improve convergence performance. The disturbance torque and its first-order derivative are treated as extended states, and a third-order disturbance observer structure is therefore established. This extended-state formulation enables simultaneous estimation of the disturbance and its dynamic variation, enhancing robustness against time-varying load perturbations. Accordingly, the state-space equations of the observer are designed as follows:

(37) $$\left\{\begin{array}{c} {\dot{\hat{\omega }}}_{e}=\frac{k_{t}p_{n}}{J}i_{q}-\frac{p_{n}}{J}{\hat{T}}_{d}+l_{1}(\omega_{e}-{\hat{\omega }}_{e})\\ {\dot{\hat{T}}}_{d}=\hat{\tau }+l_{2}(\omega_{e}-{\hat{\omega }}_{e})\\ \dot{\hat{\tau }}=l_{3}(\omega_{e}-{\hat{\omega }}_{e}) \end{array}\right.$$

where ${\hat{\omega }}_{e},{\hat{T}}_{d}$ and $\hat{\tau }$ denote the estimated values of the states $\omega_{e},T_{d}$ and τ, respectively. The parameters $l_{1},l_{2}$ and $l_{3}$ are the observer gains, which are used to regulate the convergence behavior of the estimation errors and determine the dynamic response characteristics of the observer.

### 3.6. Estimation Error Dynamics

The estimation errors are defined as follows:

(38) $$\left\{\begin{array}{c} e_{1}=\omega_{e}-{\hat{\omega }}_{e}\\ e_{2}=T_{d}-{\hat{T}}_{d}\\ e_{3}=\tau -\hat{\tau } \end{array}\right.$$

By differentiating the estimation errors and substituting the system dynamics and the observer equations, the following error dynamic equations are obtained:

(39) $$\left\{\begin{array}{c} {\dot{e}}_{1}=-\frac{p_{n}}{J}-l_{1}e_{1}\\ {\dot{e}}_{2}=\tau -(\hat{\tau }+l_{2}e_{1})=e_{3}-l_{2}e_{1}\\ {\dot{e}}_{3}=-l_{3}e_{1} \end{array}\right.$$

This can be expressed in a compact matrix form as follows:

(40) $$\left[\begin{array}{c} {\dot{e}}_{1}\\ {\dot{e}}_{2}\\ {\dot{e}}_{3} \end{array}\right]=\left[\begin{array}{ccc} -l_{1} & -\frac{p}{J} & 0\\ -l_{2} & 0 & 1\\ -l_{3} & 0 & 0 \end{array}\right]\left[\begin{array}{c} e_{1}\\ e_{2}\\ e_{3} \end{array}\right]$$

Let the error state matrix be denoted as A; then, the stability of the error system is entirely determined by the eigenvalues of A.

Accordingly, the characteristic polynomial of the error system is given by:

(41) $$det(SI-A)=det\left[\begin{array}{ccc} S+l_{1} & \frac{p_{n}}{J} & 0\\ l_{2} & S & 1\\ l_{3} & 0 & S \end{array}\right]=S^{3}+l_{1}S^{2}-\frac{p_{n}}{J}l_{2}S-\frac{p_{n}}{J}l_{3}$$

To ensure asymptotic convergence of the estimation errors to zero, the observer gains $l_{1},l_{2}$ and $l_{3}$ must be selected such that all eigenvalues of matrix A have negative real parts. Using the pole placement method, the desired pole locations are specified as $\lambda_{1},\lambda_{2}$ and $\lambda_{3}$. Accordingly, the desired characteristic polynomial is defined as:

(42) $$(S+\lambda_{1})(S+\lambda_{2})(S+\lambda_{3})=S^{3}+(\lambda_{1}+\lambda_{2}+\lambda_{3})S^{2}+(\lambda_{1}\lambda_{2}+\lambda_{1}\lambda_{3}+\lambda_{2}\lambda_{3})S+\lambda_{1}\lambda_{2}\lambda_{3}$$

By equating the two characteristic polynomials and matching the corresponding coefficients, the following relationships are obtained:

(43) $$\left\{\begin{array}{c} l_{1}=\lambda_{1}+\lambda_{2}+\lambda_{3}\\ l_{2}=-\frac{J}{p_{n}}(\lambda_{1}\lambda_{2}+\lambda_{1}\lambda_{3}+\lambda_{2}\lambda_{3})\\ l_{3}=-\frac{J}{p_{n}}\lambda_{1}\lambda_{2}\lambda_{3} \end{array}\right.$$

Since $\lambda_{I}>0$, it follows that $l_{1}$ > 0, while $l_{2}<0$, $l_{3}<0$ (with $p_{n},J$ being positive). Therefore, the observer gains must satisfy $l_{1}>0,l_{2}<0,$ and $l_{3}<0$ while also meeting the stability condition $l_{1}l_{2}<l_{3}$. Specifically,${\;l}_{1}\;$ directly affects the convergence speed of the estimated angular velocity; increasing ${\;l}_{1}$ accelerates velocity estimation but may amplify measurement noise. The gain $l_{2\;}$ influences the convergence speed of the disturbance torque estimation and determines the sensitivity of the disturbance estimate to velocity errors. Finally, $l_{3}$ governs the convergence speed of the disturbance derivative estimation, which determines the system’s ability to track rapid changes in external disturbances.

To select the values of $l_{1},l_{2}$, and ${\;l}_{3}$, the same pole placement method is applied, and the desired characteristic polynomial is specified as follows:

(44) $${\left(s+\lambda \right)}^{3}=s^{3}+3\lambda s^{2}+3\lambda^{2}s+\lambda^{3}$$

By comparing the coefficients of the polynomials, the observer gains are determined as follows:

(45) $$\left\{\begin{array}{c} l_{1}=3\lambda \\ -\frac{p_{n}}{J}l_{2}=3\lambda^{2}\\ -\frac{p_{n}}{J}l_{3}=\lambda^{3} \end{array}\right.$$

Substituting the motor parameters p_n_ = 4, J = 0.0028 $kg\cdot m^{2}$, and the desired motor speed of 50 rad/s, the observer gains can be calculated accordingly.

This yields $l_{1}=150,l_{2}=-5.25$ and ${\;l}_{3}=-262.5$, which satisfy the stability condition $l_{1}l_{2}<l_{3}$. These values represent approximate ranges; the actual gains should be fine-tuned based on the specific characteristics of the real system.

**Figure 4:**
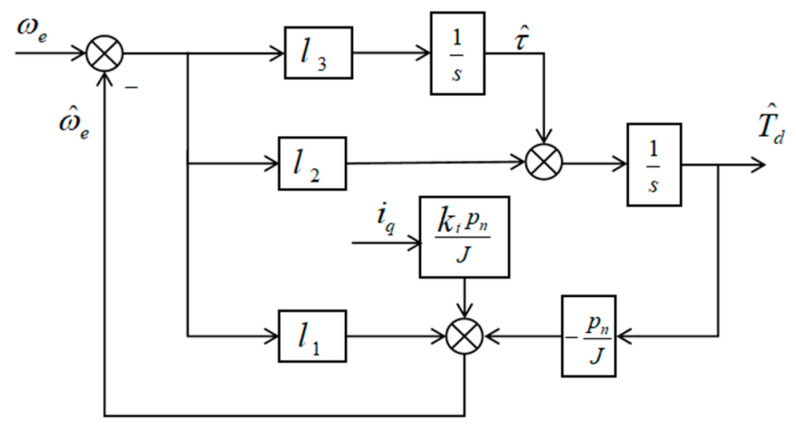
Block diagram of the HDOB structure.

The proposed high-order disturbance observer incorporates the disturbance and its derivative into the estimation process through state augmentation, and uses the speed measurement error for correction, thereby achieving real-time estimation of both the speed and the disturbance. Its structural block diagram is shown in Figure 4. Through pole placement, the convergence rate of the estimation error can be flexibly adjusted while ensuring system stability. In practical applications, the observer gains $l_{1},l_{2},$ and $l_{3}$ should be selected according to the motor parameters $p_{n}$ and J, as well as the desired dynamic performance, while also taking into account the effects of measurement noise and model uncertainties on estimation accuracy.

## 4. Simulation and Experimental Results Analysis

The effectiveness of the proposed sliding mode control incorporating the novel reaching law and the enhanced sliding-mode disturbance observer was first evaluated through simulations in MATLAB R2025a/Simulink. Table 1 lists the motor parameters used in the simulations.

### 4.1. Simulation Analysis

**Figure 5:**
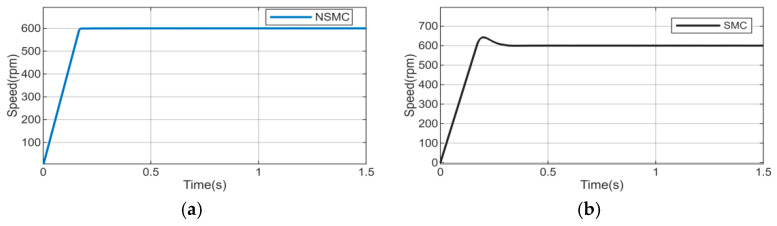
Motor speed response curve. (**a**) Speed response of NSMC at 600 r/min. (**b**) Speed response of SMC at 600 r/min.

A set of comparative simulations was designed to assess the performance of the adaptive reaching law. Specifically, a sliding mode controller based on the adaptive reaching law was compared with its counterpart based on the conventional reaching law in the speed-loop control. The parameters of the adaptive reaching law sliding mode controller were set as follows: ε=20, which determines the basic attraction strength toward the sliding surface, and a relatively large value was selected to ensure sufficient reaching capability and fast initial convergence; k = 0.7, which is used to further improve the convergence speed when the state is far from the sliding surface; α = 0.9, which determines the nonlinear variation characteristic of the power term; q = 0.1, which mainly provides a damping effect and improves the smoothness of the reaching process; λ=0.01, which was chosen to enhance the adaptive adjustment effect when the sliding variable is relatively large; $\delta_{0}=0.1$, which is the basic smoothing factor in the adaptive saturation function and determines the smoothness near the sliding surface; η=30, which controls the transition speed from the smooth region to the sign-like region; and c = 10.7, which determines the dynamic balance between the error and its derivative in the sliding surface design. The parameters of the conventional reaching law controller were set as c = 14 and q = 0.2. The simulation time was 1.5 s, and the initial reference speed was set to 600 r/min. The comparative results of the two control strategies are shown in Figure 5.

The figure reveals that the sliding mode controller employing the improved adaptive reaching law reaches the reference speed in 0.18 s with negligible overshoot, whereas the controller using the conventional reaching law achieves the target speed in 0.32 s, exhibiting a maximum speed of 642.5 rpm and an overshoot of 7.08%. These simulation results demonstrate that the proposed adaptive reaching law outperforms the conventional exponential reaching law in terms of rapidity while achieving nearly zero overshoot.

**Figure 6:**
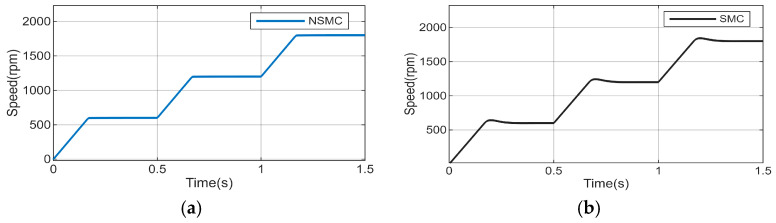
Speed step response curve. (**a**) Speed response of NSMC under sudden speed changes. (**b**) Speed response of SMC under sudden speed changes.

To further verify the dynamic stability of the proposed control approach, the reference speed is set to 600 r/min initially, stepped to 1200 r/min at 0.5 s, and then to 1800 r/min at 1 s. The simulation results are illustrated in Figure 6.

It can be observed from Figure 6 that, in terms of tracking performance after sudden changes in speed, the improved adaptive reaching law-based sliding mode controller outperforms the conventional reaching law-based sliding mode controller, and also exhibits better speed stability.

**Figure 7:**
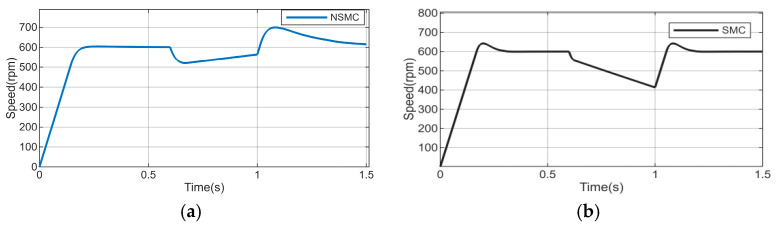
Speed response without load disturbance compensation and estimated disturbance. (**a**) Speed response of NSMC under load disturbance. (**b**) Speed response of SMC under load disturbance.

To further verify the dynamic performance of the proposed adaptive reaching law-based sliding mode control combined with a high-order disturbance observer, a disturbance torque of 1 N·m is suddenly applied at 0.6 s when the speed is 600 r/min. The parameters of the high-order disturbance observer are set as $l_{1}=200,l_{2}=-7.56,l_{3}=-250$. The disturbance torque is removed at 1 s. The simulation results are shown in Figure 7.

**Figure 8:**
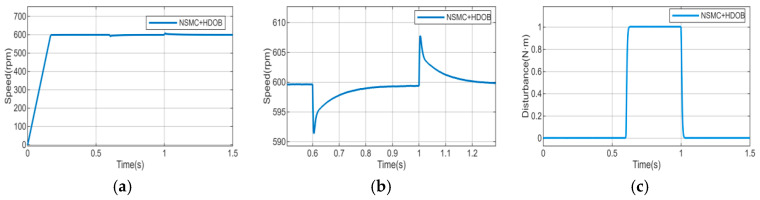
Speed response of NSMC with load disturbance compensation. (**a**,**b**) Speed response of NSMC + HDOB under load disturbance at the reference speed of 600 r/min. (**c**) Estimated disturbance of NSMC + HDOB.

**Figure 9:**
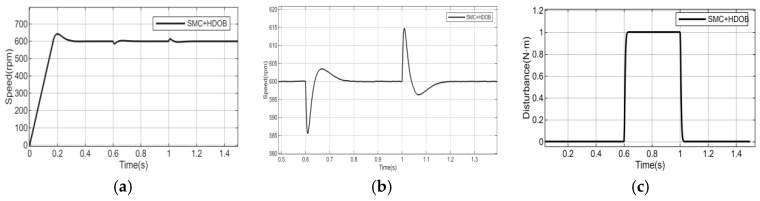
Speed response of SMC with load disturbance compensation. (**a**,**b**) Speed response of SMC + HDOB under load disturbance at the reference speed of 600 r/min. (**c**) Estimated disturbance of SMC + HDOB.

**Figure 10:**
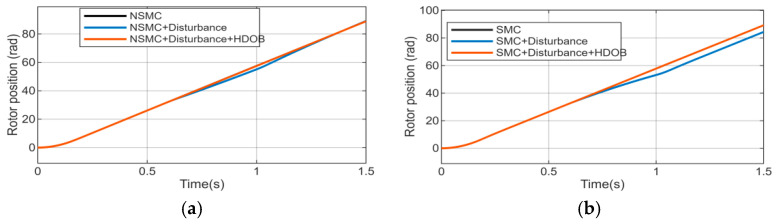
Rotor position response curve. (**a**) Rotor position response of NSMC under different operating conditions. (**b**) Rotor position response of SMC under different operating conditions.

**Figure 11:**
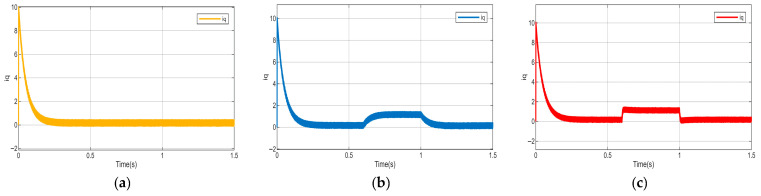
Actual iq current response of NSMC. (**a**) iq current of NSMC under normal operating conditions. (**b**) iq current of NSMC under disturbance conditions. (**c**) iq current of NSMC + HDOB under disturbance conditions.

It can be observed from Figure 7 that, under the influence of external load disturbances, the motor speed exhibits significant fluctuations, which adversely affect the normal operation of the system. To enhance the disturbance rejection capability, a high-order disturbance observer is introduced in this study. Figure 8, Figure 9, Figure 10 and Figure 11 simultaneously present the response curves of motor speed, rotor position, and reference current iq The results indicate that the introduced observer effectively improves the system’s disturbance rejection performance.

The analysis of the speed wave forms shown in Figure 8 and Figure 9 indicates that:

The conventional sliding mode control (SMC) exhibits the largest speed fluctuations. When a load disturbance is applied, the maximum speed deviation reaches 185.6 r/min, while upon disturbance removal, the maximum fluctuation is 52.4 r/min.

The adaptive sliding mode control (NSMC) shows relatively reduced but still significant speed fluctuations. The maximum speed deviation is 86 r/min during disturbance application and 105 r/min during disturbance removal.

The conventional sliding mode control combined with the high-order disturbance observer (SMC + HDOB) demonstrates improved performance. The maximum speed fluctuation is reduced to 13.1 r/min when the disturbance is applied and 14.3 r/min when the disturbance is removed.

The proposed adaptive sliding mode control combined with the high-order disturbance observer (NSMC + HDOB) achieves the best performance. The maximum speed fluctuation is further reduced to 9.1 r/min during disturbance application and 8.3 r/min during disturbance removal.

The NSMC + HDOB strategy emphasizes overshoot suppression and fluctuation reduction near the equilibrium point. Therefore, in some transient recovery processes, the return of the speed to the reference value may appear relatively smoother and less aggressive.

As shown in Figure 10, after the disturbance is applied, noticeable chattering and tracking errors appear in the rotor position response under both SMC and NSMC based control. After compensation by the HDOB, the rotor position response curves under both control strategies become smooth, and the chattering is effectively suppressed. The position tracking accuracy is significantly improved in both dynamic and steady-state processes, which verifies the effectiveness of the HDOB in enhancing disturbance rejection capability and steady-state accuracy.

To further verify the effectiveness of the proposed control strategy, The experimental results are shown in Figure 11, under disturbance-free conditions, NSMC enables the current iq to accurately track its reference with satisfactory performance. However, when external disturbances are present, significant steady-state errors and dynamic performance degradation occur in the current response, leading to reduced control performance. With the introduction of the HDOB for disturbance estimation and feedforward compensation, the current rapidly recovers and achieves accurate tracking.

**Figure 12:**
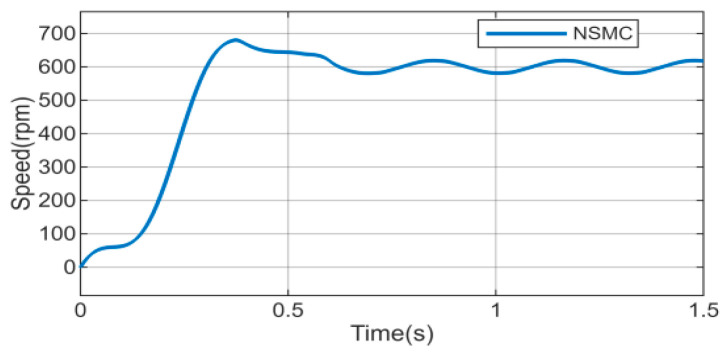
Speed response of NSMC under motor parameter variations and external disturbances.

**Figure 13:**
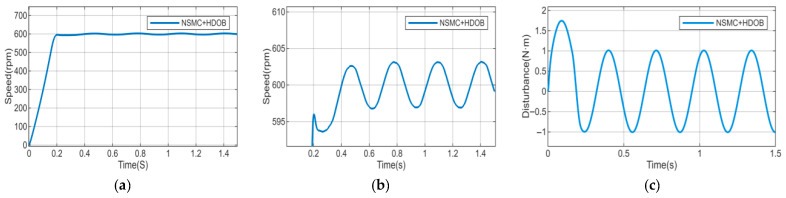
(**a**,**b**) Speed responses of NSMC + HDOB under motor parameter variations and external disturbances. (**c**) Estimated external disturbance of NSMC + HDOB.

**Figure 14:**
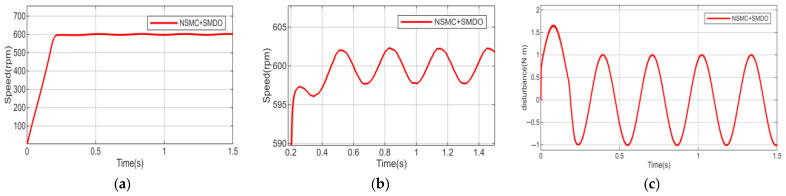
(**a**,**b**) Speed responses of NSMC + SMDO under motor parameter variations and external disturbances. (**c**) Estimated external disturbance of NSMC + SMDO.

Considering the diversity of disturbance types, to further validate the disturbance rejection capability of the proposed algorithm, the external disturbance is replaced by a sinusoidal function with an amplitude of 1 and a frequency of 20 rad/s. Meanwhile, to simulate parameter variations caused by long-term motor operation, the motor parameters (inductance, stator resistance, and moment of inertia) are set to 1.5 times their nominal values. Under these conditions, a comparative study with the sliding mode disturbance observer is conducted, and the results are shown in Figure 12, Figure 13 and Figure 14.

As shown in Figure 12, when external load disturbances and motor parameters vary, the motor speed under NSMC control exhibits large fluctuations and poor tracking performance, which adversely affects normal operation. After introducing the HDOB or SMDO observer (Figure 13 and Figure 14), the operating condition of the motor is significantly improved. In terms of disturbance estimation, the estimation curve of the HDOB is smoother than that of the SMDO, without noticeable spikes, indicating that the HDOB provides more accurate disturbance compensation capability.

Based on the above simulation results, the NSMC + HDOB control strategy demonstrates superior overall performance. It achieves fast and accurate speed tracking, enabling precise tracking of the reference speed without overshoot. Moreover, it exhibits strong robustness against disturbances. Under load disturbances, it can quickly and accurately estimate disturbances and compensate them in real time, effectively suppressing speed fluctuations and improving the overall transient behavior. It should be noted that the proposed adaptive reaching law intentionally reduces the switching intensity when the system state approaches the sliding surface. Therefore, although the recovery process may appear slightly smoother in some transient cases, this design helps suppress overshoot and chattering and provides a better trade-off among recovery speed, fluctuation reduction, and robustness.

### 4.2. Experimental Analysis

**Figure 15:**
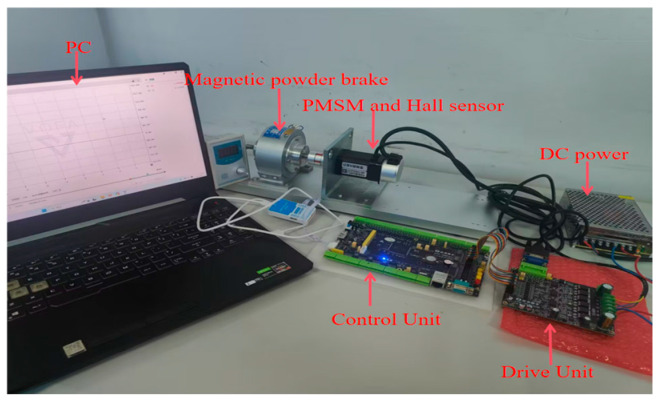
Experimental setup.

Real-time experiments were conducted on a PMSM drive system to further verify the effectiveness of the proposed control strategy. The NSMC + HDOB method was implemented for PMSM speed regulation on an STM32F407IGT6 digital control board using C programming; the experimental platform is depicted in Figure 15. A three-phase PWM inverter employing an intelligent power module (IPM) served as the motor driver, operating at a switching frequency of 10 kHz. A magnetic powder brake was employed to provide load torque, with the load magnitude adjustable through the excitation current. In addition, rotor position feedback was obtained from three Hall position signals (PHu, PHv, and PHw), and the rotor speed was calculated by the controller based on the time interval between two adjacent Hall signal transitions.

To evaluate the control performance, experiments were first conducted to compare the proposed NSMC with conventional SMC. For a fair comparison, the PI parameters of the current inner loop were kept identical, and the controller parameters of NSMC and SMC were selected to be the same as those used in the simulations.

**Figure 16:**
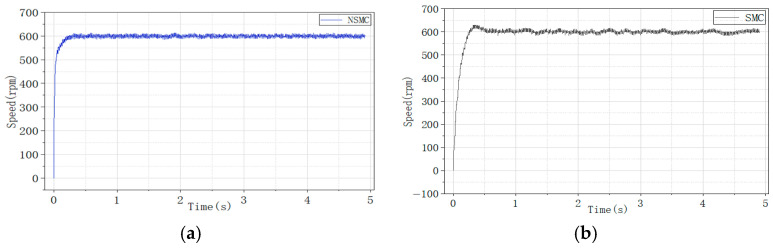
Speed comparison between NSMC and SMC. (**a**) Motor speed response under NSMC. (**b**) Motor speed response under SMC.

The system performance under NSMC and conventional SMC was first compared. The PI parameters for both current loops were kept identical, and the controller parameters for NSMC and SMC were set the same as those used in the simulations. Since the PMSM is influenced by environmental factors during actual operation, the experimental results are expected to differ from the simulation data. The corresponding experimental results are shown in the following Figure 16.

The experimental results indicate that the conventional sliding mode controller reaches the target speed in 0.5011 s, with a maximum speed of 630 r/min, corresponding to an overshoot of 5%. In contrast, the proposed adaptive sliding mode controller reaches the target speed in 0.2849 s with almost no overshoot. These results further confirm the fast convergence and low oscillation characteristics of the proposed adaptive reaching law.

**Figure 17:**
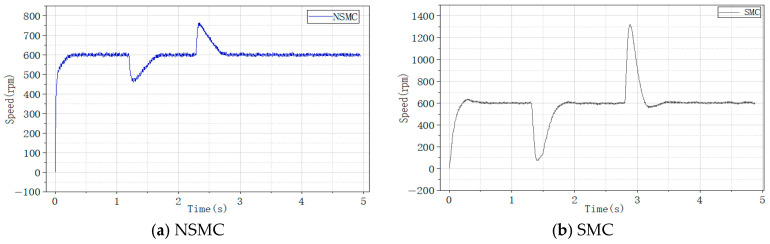
Comparison of speed responses between NSMC and SMC under load disturbance. (**a**) Speed response of NSMC under load disturbance; (**b**) speed response of SMC under load disturbance.

To further validate the performance of the proposed high-order disturbance observer, a load disturbance of 1 N·m was applied to the motor using the magnetic powder brake. In this experiment, the brake excitation current was set to 0.04 A to generate the disturbance, as shown in Figure 17.

The experimental results show that when a load disturbance is applied, the proposed adaptive sliding mode controller exhibits smaller speed fluctuations compared to the conventional sliding mode controller. However, the speed variations are still significant enough to affect the normal operation of the motor.

**Figure 18:**
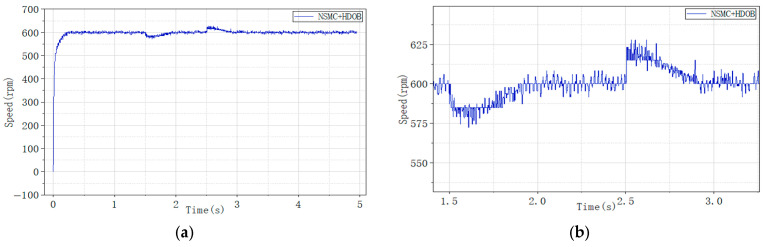
(**a**) Motor speed response under NSMC + HDOB control. (**b**) Enlarged view of motor speed response under NSMC + HDOB control.

**Figure 19:**
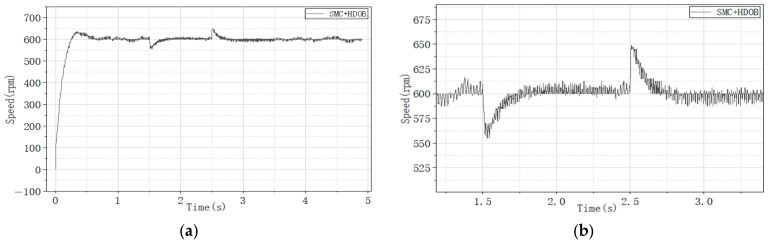
(**a**) Motor speed response under SMC + HDOB control. (**b**) Enlarged view of motor speed response under SMC + HDOB control.

**Figure 20:**
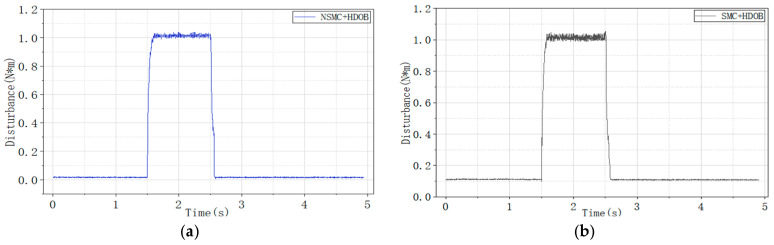
Disturbance torque estimated by the HDOB. (**a**) Disturbance estimation under NSMC + HDOB control. (**b**) Disturbance estimation under SMC + HDOB control.

To further enhance disturbance rejection, a high-order disturbance observer (HDOB) is integrated with both control strategies. The corresponding experimental results are illustrated in Figure 18, Figure 19 and Figure 20, where Figure 18 shows the motor speed response under NSMC + HDOB control, Figure 19 shows the response under SMC + HDOB control, and Figure 20 depicts the estimated disturbance torque.

To evaluate the effectiveness of the proposed control strategy, comparative experiments were carried out under sudden load application and removal conditions to compare the performance of conventional sliding mode control with a high-order disturbance observer (SMC + HDOB) and the proposed adaptive sliding mode control with a high-order disturbance observer (NSMC + HDOB).

The experimental results indicate that both control strategies can achieve speed tracking; however, the NSMC + HDOB exhibits improved disturbance attenuation capability and smoother dynamic response under dynamic disturbances. With the SMC + HDOB strategy, the maximum speed deviation during load application reaches 45 r/min, and at the moment of load removal, it rises to 64.87 r/min. In contrast, the proposed NSMC + HDOB strategy demonstrates outstanding disturbance rejection capability: under the same conditions, the maximum speed deviation during load application is significantly reduced to 27.76 r/min, and only 27.75 r/min when the load is removed.

In summary, the NSMC + HDOB control strategy demonstrates significant overall performance advantages:

Fast, overshoot-free tracking: The strategy enables rapid and precise tracking of the reference speed command without any overshoot during transients, thereby improving the accuracy of speed tracking.

Strong disturbance rejection and robustness: Thanks to the real-time, accurate estimation and compensation provided by the high-order disturbance observer, the strategy effectively suppresses speed fluctuations under load disturbances.

These performance improvements are primarily attributed to two factors. First, the sliding mode control design based on the proposed adaptive reaching law improves the smoothness of the control action and effectively reduces control-induced oscillations, thereby minimizing overshoot and enhancing tracking accuracy. It should be noted that the adaptive reaching law intentionally reduces the convergence rate near the sliding surface, resulting in a smoother but slightly more gradual dynamic response. Second, the synergy between the high-order disturbance observer and sliding mode control enables accurate estimation of aggregated disturbances and real-time feedforward compensation, which significantly enhances the system’s disturbance rejection capability and robustness.

## 5. Discussion

This paper presents a composite speed control strategy for permanent magnet synchronous motor (PMSM) that integrates a novel adaptive reaching law-based sliding mode control with a high-order disturbance observer. To resolve the trade-off between convergence speed and chattering suppression, and to overcome the limited disturbance rejection capability of conventional sliding mode control during speed regulation, a new adaptive reaching law is developed along with an adaptive saturation function. This design accelerates the approaching process when the system state is distant from the sliding surface while reducing switching intensity near the equilibrium point, thereby enhancing dynamic response and minimizing chattering. On this basis, a high-order disturbance observer is employed to estimate the load disturbance and its rate of change online, with the estimated value fed forward to the output of the speed-loop controller, further improving disturbance rejection and system robustness. The stability of the proposed method is verified using Lyapunov theory. Simulation and experimental results demonstrate that, compared with conventional sliding mode control and its observer-compensated counterpart, the proposed NSMC + HDOB method achieves faster response, reduced speed fluctuations, and enhanced robustness against load disturbances. This approach offers a valuable reference for the control design of high-performance PMSM drive systems.

## Figures and Tables

**Table 1:** Motor Parameters.

Parameter Name	Parameter Value
Number of Pole Pairs $p_{n}$	4
Inductance $L_{s}/mH$	0.59
Stator Resistance R/Ω	1.02
Flux Linkage $\varphi_{f}/V/krpm$	4.3
Rotor Inertia $J/kg\cdot m^{2}$	0.0028

## Data Availability

The data are contained within the article.
